# Re-evaluating the lying-down test: a step-saving and well-tolerated diagnostic adjunct for horizontal canal benign paroxysmal positional vertigo

**DOI:** 10.3389/fneur.2026.1830444

**Published:** 2026-05-25

**Authors:** Kai Xia, Rui Gao, Xiaodi Zhang, Xiaoxiao Yan, Dandan He

**Affiliations:** 1 Department of Neurology, The Third Affiliated Hospital of Wenzhou Medical University, Wenzhou, China; 2 Department of Neurology, Changzheng Hospital, Navy Medical University, Shanghai, China; 3 Department of Neurology, Shijiazhuang People’s Hospital (Jianhua Campus), Shijiazhuang, China; 4 Department of Pharmacy, The Third Affiliated Hospital of Wenzhou Medical University, Wenzhou, China

**Keywords:** benign paroxysmal positional vertigo, diagnosis, horizontal canal BPPV, lying-down test, nystagmus

## Abstract

**Background:**

The supine roll test (SRT) is the established diagnostic standard for horizontal canal benign paroxysmal positional vertigo (HC-BPPV). However, its clinical application is constrained by variable nystagmus patterns and significant patient discomfort during the maneuver. The lying-down test (LDT) has been suggested as a more tolerable and simpler alternative, though its diagnostic performance remains inconsistently reported in the literature. This study systematically evaluates the diagnostic value of LDT for HC-BPPV.

**Methods:**

This retrospective analysis included 209 patients with definitively diagnosed HC-BPPV. All participants underwent standard positional testing with comprehensive recording of nystagmus characteristics, including direction, intensity, latency, duration, and affected-side determination. Following diagnosis, all patients were successfully treated with canalith repositioning therapy. Participants were stratified into two groups based on their fLDT (first LDT) result: fLDT-positive and fLDT-negative. Comparative analyses of nystagmus parameters and diagnostic efficiency were conducted between groups. Logistic regression was employed to identify factors associated with a positive LDT response.

**Results:**

The overall positive rate of LDT was 60.3%, with a side-determination accuracy of 96.7%. The first LDT (fLDT) positive rate was 44.5%. Among canalithiasis patients, the fLDT-positive group demonstrated superior diagnostic efficiency, requiring fewer diagnostic steps than the fLDTnegative group (z = −4.138, *p* < 0.001), and also exhibited significantly greater intensity (FLNI, *p* = 0.003) and shorter latency (FLNL, *p* = 0.003) of the nystagmus induced by the first leftward roll. In cupulolithiasis patients, only shorter FLNL was associated with a positive fLDT (*p* = 0.031). After adjusting for confounders, logistic regression indicated that older age (≥60 years) was an independent predictor of positive LDN in canalithiasis HC-BPPV (OR = 2.245, 95% CI: 1.176–4.286, *p* = 0.014), an association not observed in cupulolithiasis.

**Conclusion:**

The LDT is a reliable and well-tolerated diagnostic tool for HC-BPPV. Its routine integration into clinical practice as an adjunct to SRT is recommended. Furthermore, this study identifies advanced age (≥60 years) as a significant predictor of positive LDT response in canalithiasis HC-BPPV, with nystagmus intensity and latency serving as key discriminators. These findings refine the understanding of LDT’s diagnostic utility and provide a foundation for further investigation into the pathophysiology of positional nystagmus in HC-BPPV.

## 1 Introduction

Benign paroxysmal positional vertigo (BPPV) is a prevalent peripheral vestibular disorder, characterized by transient episodes of vertigo and distinctive nystagmus triggered by specific changes in head position relative to gravity. Based on the affected semicircular canal, BPPV is classified into posterior canal BPPV (PC-BPPV, the most common type), horizontal canal BPPV (HC-BPPV), the rare anterior canal BPPV (AC-BPPV), and multicanal BPPV (1–3). Among these, HC-BPPV constitutes the second most frequent form, representing approximately 16–31% of all BPPV cases (4). HC-BPPV can be further divided into two distinct pathophysiological subtypes: canalithiasis and cupulolithiasis. Canalithiasis occurs when otoconia detached from the utricular macula become free-floating within the semicircular canal lumen; their movement under gravity induces endolymph flow, leading to abnormal cupular deflection. In contrast, cupulolithiasis results from otoconia adhering directly to the cupula of the ampulla, thereby altering its density relative to the surrounding endolymph and rendering it gravity-sensitive.

Diagnosis of BPPV relies fundamentally on positional testing, which is essential for accurate identification and subsequent management. For PC-BPPV, the Dix-Hallpike maneuver is the standard diagnostic test. However, diagnosing HC-BPPV poses a greater clinical challenge, primarily due to difficulties both in eliciting the characteristic nystagmus and in accurately determining the affected side (5). Currently, the supine roll test (SRT) — also known as the McClure-Pagnini test or supine head yaw test (SHYT) — is widely considered the gold standard for lateralizing HC-BPPV and distinguishing between its subtypes (canalithiasis vs. cupulolithiasis). This test involves rapidly rotating the patient’s head to each side while in the supine position (often with 30° head elevation), observing the induced horizontal nystagmus. Analysis of nystagmus direction, intensity, and duration then guides identification of the pathologic side and underlying mechanism, informing appropriate repositioning therapy (5).

In clinical practice, however, nystagmus patterns in HC-BPPV can be highly variable and complex, frequently complicating the determination of the affected canal and thus impeding effective treatment (6). To address these limitations, several alternative or supplementary positional tests have been explored, including the bowandlean test (BLT), pseudo-spontaneous nystagmus (PSN), and LDT (7–9). Nevertheless, existing studies suggest that such tests often exhibit relatively low rates of eliciting diagnostic nystagmus and may produce nystagmus of weaker intensity, limiting their definitive clinical utility and underscoring the need for further validation (7–9).

Among alternative diagnostic maneuvers, the lying-down test (LDT) has gained considerable international attention due to its procedural simplicity—requiring only a rapid transition from sitting to supine—and its favorable patient tolerance. First described by Nuti et al. (10) and also termed seated-supine positioning nystagmus (SSPN), LDT is conventionally understood to elicit nystagmus beating toward the healthy side in canalithiasis HC-BPPV and toward the affected side in cupulolithiasis HC-BPPV (11).

Despite these theoretical and practical advantages, recent investigations have reported marked inconsistencies in the elicitation rate, nystagmus characteristics, and clinical utility of LDT. For instance, Han et al. (11) observed LDN in only 38.2% of 152 HC-BPPV patients, whereas Asprella-Libonati (12) reported a rate as high as 96%. Subsequent studies further reflect this variability: Yetiser et al. (13) documented an SSPN rate of 57.7%; Liang et al. (14) reported 73.3% in 200 patients; Wu et al. (15) noted a rate of only 30.0%; and Oh et al. (8) found SSPN in 68% of cases. Moreover, Oh et al. (8) questioned the lateralizing value of SSPN, as nystagmus beat toward the healthy side in only 61.3% of canalithiasis cases and toward the affected side in merely 78.9% of cupulolithiasis cases.

Given these persistent controversies regarding its diagnostic performance and lateralization reliability, the present study aims to systematically evaluate the clinical value of LDT in HC-BPPV by analyzing its positivity rate, accuracy in identifying the affected side, and the detailed features of elicited nystagmus. Furthermore, we seek to explore potential mechanisms underlying the generation of LDN.

## 2 Methods

### 2.1 Patient selection and data collection

This retrospective study analyzed outpatient data from Shanghai Changzheng Hospital between October 1, 2024 and September 30, 2025. A total of 218 patients diagnosed with HC-BPPV were initially identified. The study protocol was approved by the Institutional Review Board of Shanghai Changzheng Hospital. The diagnosis of BPPV was established according to the diagnostic criteria outlined in the International Classification of Vestibular Disorders (ICVD) guidelines published by the Bárány Society in 2015 (2). Inclusion criteria were as follows: (1) meeting the 2015 Bárány Society diagnostic criteria for BPPV; (2) clear lateralization (determination of the affected side) and identification of the subtype (canalithiasis or cupulolithiasis) achievable via both the Supine Roll Test and the Lying-Down Test; (3) documented effectiveness of canalith repositioning maneuvers. Exclusion criteria included: (1) presence of spontaneous nystagmus; (2) multi-canal BPPV; (3) concurrent other inner ear diseases; (4) presence of central nervous system disorders; (5) inability to complete the positional testing.

### 2.2 Diagnosis and treatment process

All diagnostic examinations and therapeutic maneuvers were conducted using a fully automated BPPV diagnosis and treatment system (the ZT-CHAIR-I rotational chair system). A key component of the system was its integrated video-oculography (VOG) goggle assembly, which was used to capture, record, and quantitatively analyze all nystagmus parameters in real-time. Based on the operator’s clinical judgment, the Dix-Hallpike test or the SRT was performed initially. Notably, the LDT was integrated as the first step of the SRT protocol. Among the 209 HC-BPPV patients, 45 underwent the Dix-Hallpike test first to rule out posterior canal BPPV, followed by the SRT. The remaining 164 patients proceeded directly to the SRT.

The SRT procedure was standardized as follows: It commenced with the LDT (patient rapidly moving from sitting to supine). Immediately following this, the patient was rolled 90° to the left, returned to the supine position, then rolled 90° to the right, and finally returned to supine, with each position maintained for at least 15 s. The determination of the affected side relied on the operator’s expertise, which could involve selectively repeating the above steps until a conclusive judgment was made. Once the affected side was confirmed, immediate repositioning treatment was administered according to the subtype. For geotropic HC-BPPV (canalithiasis), patients received one session of Barbecue maneuver followed by one session of Gufoni maneuver (healthy side, head 45° downward). For apogeotropic HC-BPPV (cupulolithiasis), patients first underwent ZT chair 360° rotation in supine to convert nystagmus to geotropic, followed by the same Barbecue and Gufoni protocol. All patients were reassessed 30 min post-treatment. If symptoms persisted, another ZT chair rotation session was performed. If asymptomatic, they were discharged with instructions to return for free retreatment within one week if symptoms recurred.

Throughout the diagnostic process, the direction, intensity, latency, and duration of the induced nystagmus were observed and recorded. It is important to note that in cases where bilateral nystagmus exhibited ambiguous intensity changes, making lateralization difficult, operators might perform multiple selective maneuvers (repeated LDT, left or right 90° rolls). For the purpose of this study, we quantified the number of diagnostic steps within the SRT: the initial LDT was counted as 1 step, each subsequent 90° roll to the left or right was counted as 1 step, while the return-to-center actions and the repositioning treatment phase itself were not counted. Any prior Dix-Hallpike tests were also excluded from this step count.

For the purpose of evaluating diagnostic accuracy, we also defined a simplified fixed-protocol index test based solely on the LDT combined with the first leftward roll, without repetition. The side was determined as follows: when LDT induced right-beating nystagmus, geotropic (left-beating) nystagmus on the first leftward roll indicated left canalolithiasis, and apogeotropic (right-beating) nystagmus indicated right cupulolithiasis; when LDT induced left-beating nystagmus, geotropic (right-beating) nystagmus on the first leftward roll indicated right canalolithiasis, and apogeotropic (left-beating) nystagmus indicated left cupulolithiasis. The final clinical diagnosis (reference standard) was made by experienced clinicians based on comprehensive assessment, including LDT, repeated maneuvers as needed, and treatment response. The accuracy of the simplified index test was calculated by comparing its side determination to this reference standard.

### 2.3 Nystagmus recording

Nystagmus parameters were captured and recorded using a video-oculography system. The following parameters were documented: Direction: Horizontal nystagmus was classified as either geotropic (beating towards the ground) or apogeotropic (beating away from the ground). Latency: The time interval, measured in seconds (s), from the completion of the positional change to the onset of observable horizontal nystagmus. Duration: The total time, measured in seconds (s), from the onset to the complete cessation of the horizontal nystagmus. Intensity: Defined as the peak slow-phase velocity (SPV) of the nystagmus, measured in degrees per second (°/s).

### 2.4 Statistical analysis

Data analysis was performed using SPSS software (version 25.0). Continuous variables that followed a normal distribution are presented as mean ± standard deviation (SD), and comparisons between groups were conducted using the independent samples t-test. Data with a non-normal distribution are expressed as median (interquartile range, IQR), and the Wilcoxon rank-sum test was used for between-group comparisons. Categorical variables are summarized as frequency (percentage), and group differences were assessed using the chi-square (χ^2^) test. Binary logistic regression analysis was employed to identify independent factors associated with a positive LDT result. Relevant variables were included in a multivariate analysis model. A two-tailed *p*-value of < 0.05 was considered statistically significant.

## 3 Results

### 3.1 Demographic characteristics

**Figure 1:**
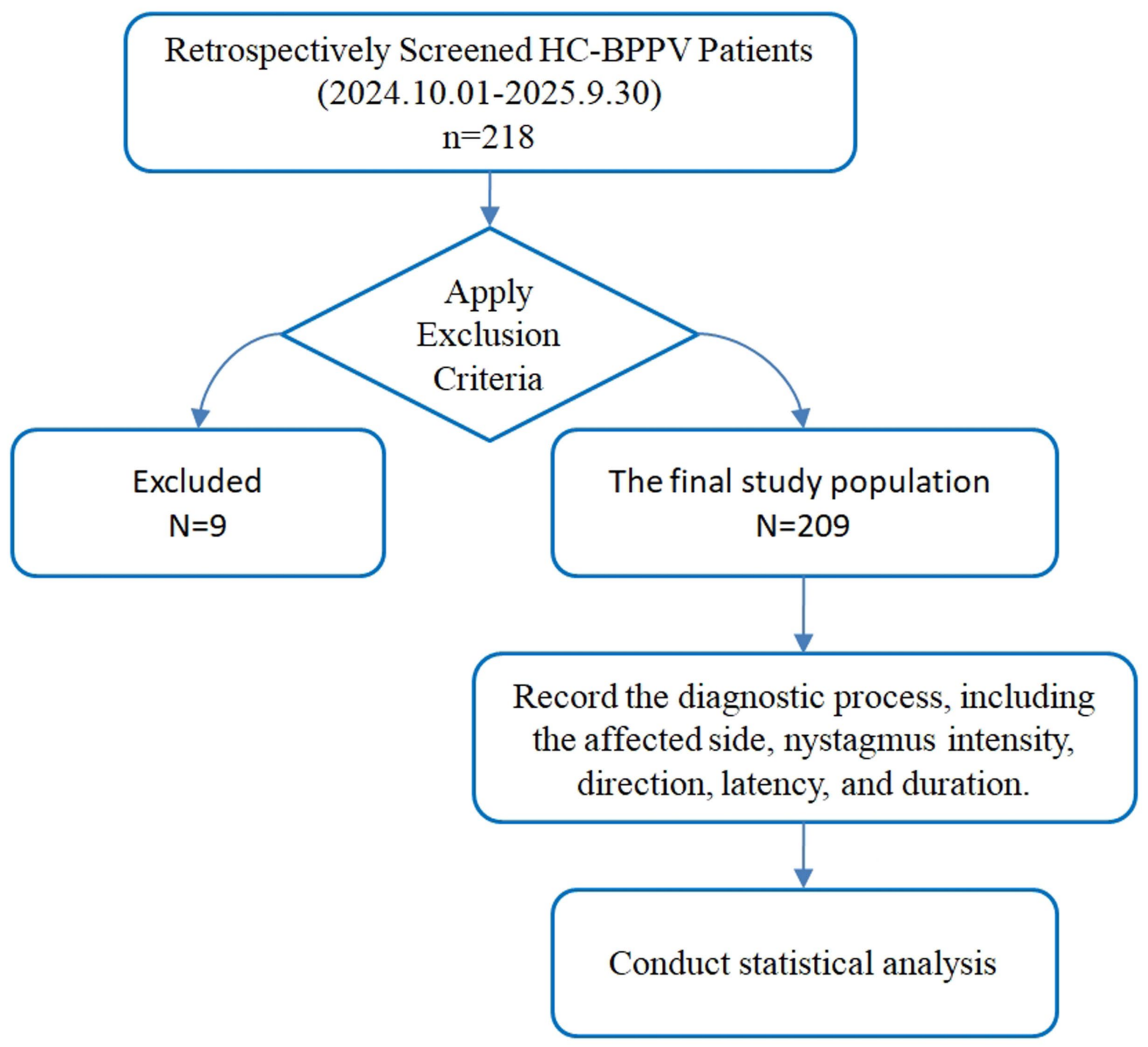
Flow chart indicating case enrollment.

A total of 218 patients diagnosed with HC-BPPV were initially identified. Nine patients were subsequently excluded due to multi-canal involvement, spontaneous nystagmus, or ineffective repositioning therapy. Thus, the final analysis included 209 patients, as depicted in the flow chart (Figure 1). The age of the cohort ranged from 23 to 90 years, with a mean age of 56.97 ± 15.21 years. Female patients (*n* = 132) outnumbered male patients (*n* = 77), yielding a female-to-male ratio of 1.71:1. Among the included patients, 162 (77.5%) were diagnosed with canalithiasis HC-BPPV and 47 (22.5%) with cupulolithiasis HC-BPPV.

The overall rate of eliciting LDN during the diagnostic process was 60.3%, with subtype-specific rates of 54.9% in canalithiasis and 78.7% in cupulolithiasis. Based on successful repositioning outcomes, the overall accuracy of LDT for lateralizing the affected side was 96.7–96.9% for canalithiasis and 95.7% for cupulolithiasis.

Participants were divided into two groups depending on whether horizontal nystagmus was induced during their first-lying test (fLDT): the fLDT-positive group (nystagmus present) and the fLDT-negative group (nystagmus absent). The positivity rate of the fLDT was 44.5% (*n* = 93), with rates of 40.1% for canalithiasis and 59.6% for cupulolithiasis. Among the entire cohort, 45 patients initially underwent the Dix-Hallpike test prior to the LDT/SRT sequence. During the standardized SRT protocol, 36 patients (17.2%) showed no nystagmus on the first 90° leftward roll.

No statistically significant differences in gender or affected side were observed between the groups. However, a significant difference was noted among age groups (*p* = 0.038), as presented in Table 1.

**Table 1:** Demographic characteristics in fLDT(+) vs. fLDT(−).

Variables	Canalithiasis (*n* = 162)		Cupulolithiasis (*n* = 47)			
	fLDT(+)	fLDT(−)	fLDT(+)	fLDT(−)	χ^2^	*p*-value
Gender, *n* (%)					2.394	0.495
Male (1)	28 (43.1)	33 (34.0)	11 (39.3)	5 (26.3)		
Female (0)	37 (56.9)	64 (66.0)	17 (60.7)	14 (73.7)		
Age Group, *n* (%)					8.402	0.038*
< 60 years (0)	27 (41.5)	60 (61.9)	15 (53.6)	7 (36.8)		
≥ 60 years (1)	38 (58.5)	37 (38.1)	13 (46.4)	12 (63.2)		
Affected Side, *n* (%)					0.658	0.883
Left (1)	28 (43.1)	45 (46.4)	12 (42.9)	7 (36.8)		
Right (2)	37 (56.9)	52 (53.6)	16 (57.1)	12 (63.2)		

fLDT, first lying-down test; *p <0.05 for the four-group comparison.

### 3.2 Comparison of diagnostic steps required in fLDT(+) vs. fLDT(−) patients

Among patients with canalithiasis BPPV, the number of diagnostic steps required was significantly lower in the fLDT(+) group compared to the fLDT(−) group, with the difference being statistically significant (z = −4.138, *p* < 0.001). In contrast, among patients with cupulolithiasis BPPV, no statistically significant difference in the number of diagnostic steps was observed between the fLDT(+) and fLDT(−) groups (z = −1.322, *p* = 0.186). The detailed data are presented in Table 2.

**Table 2:** Comparison of diagnostic steps in fLDT(+) vs. fLDT(−).

Subtype	*N* = 209	fLDT(+) (*n* = 93), Mean (SD)	fLDT(−) (*n* = 116), Mean (SD)	Z value	*p*-value
Canalithiasis	162	4.06 (1.88)	5.25 (2.126)	−4.138	<0.001*
Cupulolithiasis	47	5.43 (1.69)	6.21 (2.275)	−1.322	0.186

fLDT, first lying-down test; SD, standard deviation. Data are presented as mean (SD). The number of diagnostic steps was compared between groups using the Wilcoxon rank-sum test. *p <0.05 indicates statistical significance.

### 3.3 Nystagmus analysis: fLDT(+) vs. fLDT(−)

Among patients with canalithiasis BPPV, the intensity of the first leftward-roll nystagmus (FLNI) was significantly greater in the fLDT(+) group than in the fLDT(−) group (*p* = 0.003). In contrast, no statistically significant difference was observed in the maximum nystagmus intensity (MaxNI) between the two groups.

For patients with cupulolithiasis BPPV, no significant differences were found between the fLDT(+) and fLDT(−) groups in either FLNI (*p* = 0.712) or MaxNI (*p* = 0.737). Detailed results are presented in Table 3.

**Table 3:** Comparison of nystagmus intensity in fLDT(+) vs. fLDT(−) by subtype.

Parameters	Canalithiasis (*n* = 162)			Cupulolithiasis (*n* = 47)		
	fLDT(+), *n* = 65	fLDT(−), *n* = 97	*p*-value	fLDT(+), *n* = 28	fLDT(−), *n* = 19	*p-*value
FLNI	24.20 (9.50, 39.85)	13.00 (1.20, 25.50)	0.003*	17.20 (6.95, 30.83)	11.70 (5.20, 35.70)	0.712
MaxNI	103.00 (56.40, 166.35)	93.90 (42.90, 154.25)	0.388	111.40 (66.58, 147.10)	97.80 (62.60, 180.00)	0.737

fLDT, first lying-down test; FLNI, first leftward-roll nystagmus intensity; MaxNI, maximum nystagmus intensity. Data are presented as median (interquartile range). The Wilcoxon rank-sum test was used for group comparisons. *p-value indicates statistical significance.

It should be noted that nystagmus was not elicited during the first 90° leftward roll in some patients, resulting in missing latency data for this specific maneuver. After excluding these cases, subsequent analysis revealed significant differences in the latency of the first leftward-roll nystagmus (FLNL) between the fLDT(+) and fLDT(−) groups, irrespective of BPPV subtype. The differences were statistically significant for both canalithiasis (*p* = 0.003) and cupulolithiasis (*p* = 0.031), as detailed in Table 4.

**Table 4:** Comparison of nystagmus latency in fLDT(+) vs. fLDT(−) by subtype.

Parameters	Canalithiasis (*n* = 129)			Cupulolithiasis (*n* = 44)		
	fLDT(+), *n* = 56	fLDT(−), *n* = 73	*p*-value	fLDT(+), *n* = 26	fLDT(−), *n* = 18	*p-*value
FLNL	0.15 (0.00, 1.48)	1.00 (0.30, 2.45)	0.003^a^	2.90 (0.68, 4.63)	0.90 (0.00, 1.48)	0.031^b^

fLDT, first lying-down test; FLNL, first leftward-roll nystagmus latency; IQR, interquartile range. Data are presented as median (IQR) in seconds (s). Group comparisons were performed using the Wilcoxon rank-sum test.

a *p* < 0.05 for canalithiasis fLDT(+) vs. fLDT(−).

b *p* < 0.05 for cupulolithiasis fLDT(+) vs. fLDT(−).

### 3.4 Logistic regression analysis: fLDT(+) vs. fLDT(−)

Patients were stratified by BPPV subtype (canalithiasis vs. cupulolithiasis) for separate logistic regression analyses. The models included the following potential predictors: gender, affected side, prior performance of a Dix-Hallpike test, and age group (dichotomized as <60 years vs. ≥60 years).

The results demonstrated that within the canalithiasis subgroup, age group was a statistically significant independent predictor of a positive fLDT result. Patients aged 60 years or older had significantly higher odds of being fLDT-positive compared to those under 60 years (OR = 2.245, 95% CI: 1.176–4.286, *p* = 0.014). None of the other variables, including gender, affected side, or prior Dix-Hallpike testing, showed a significant association with fLDT status in either the canalithiasis or cupulolithiasis models. The detailed regression coefficients and statistics are presented in Table 5.

**Table 5:** Logistic regression analysis: fLDT(+) vs. fLDT(−).

Subtype and variable	b (SE)	Wald χ^2^	*p*-value	OR (95% CI)
Canalithiasis				
Gender (male vs. female)	0.356 (0.344)	1.076	0.300	1.428 (0.728–2.800)
Side (left vs. right)	0.218 (0.338)	0.416	0.519	1.243 (0.641–2.410)
Prior Dix-Hallpike (yes vs. no)	−0.035 (0.408)	0.008	0.931	0.965 (0.434–2.147)
Age group (≥60 vs. <60 years)	0.809 (0.330)	6.003	0.014*	2.245 (1.176–4.286)
Cupulolithiasis				
Gender (male vs. female)	0.506 (0.678)	0.557	0.455	1.659 (0.439–6.269)
Side (left vs. right)	0.063 (0.659)	0.009	0.924	1.065 (0.293–3.877)
Prior Dix-Hallpike (yes vs. no)	0.631 (0.779)	0.657	0.418	1.880 (0.408–8.661)
Age group (≥60 vs. <60 years)	−0.635 (0.631)	1.014	0.314	0.530 (0.154–1.824)

b, regression coefficient; SE, standard error; OR, odds ratio; CI, confidence interval; fLDT, first lying-down test. *p < 0.05. After adjusting for gender, affected side, and prior Dix-Hallpike testing, age group remained a significant independent predictor of fLDT positivity in the canalithiasis subtype.

## 4 Discussion

This study demonstrates that the LDT is a reliable and well-tolerated diagnostic method for HC-BPPV. Although its overall positivity rate (60.3%) and the rate of the first LDT (fLDT, 44.5%) show some variability, the test achieves a notably high lateralization accuracy of 96.7%, underscoring its clinical utility. Importantly, this is the first study to quantify “diagnostic steps,” revealing that a positive fLDT can significantly streamline the diagnostic process for canalithiasis HC-BPPV by reducing the number of required maneuvers, thereby optimizing clinical workflow and improving patient comfort.

Our results align with most previous reports, further validating the diagnostic effectiveness of LDT in HC-BPPV. We also introduced and evaluated the concept of fLDN. The fLDT positivity rates were 40.1% for canalithiasis and 59.6% for cupulolithiasis, with the latter’s higher rate consistent with prevailing understanding. The horizontal semicircular canal comprises anterior and posterior arms. In canalithiasis, if otoconia are situated in the posterior arm when the patient is seated, the rapid recline during LDT causes the particles to move away from the ampulla due to gravity, inhibiting the affected labyrinth and producing nystagmus beating toward the healthy side. Conversely, if otoconia are located in the anterior arm, LDT may induce movement toward the ampulla, exciting the affected labyrinth and potentially leading to nystagmus beating toward the affected side, which could result in erroneous lateralization. The high lateralization accuracy observed in our study, combined with the shorter anatomical course of the anterior arm, supports the hypothesis that otoconia in most HC-BPPV patients are located in the posterior arm. Hence, a positive LDN holds substantial value for accurate lateralization in HC-BPPV.

However, the overall LDT positivity rate in our study was 60.3%, leaving a considerable proportion of patients with negative results. A recent simulation study by Bhandari et al. (16) demonstrated that the initial position of otoconia within the horizontal canal significantly affects nystagmus patterns during the supine roll test (SRT). Extrapolating from their findings, we hypothesize that this factor may also influence whether LDT elicits nystagmus. For example, if debris is already located near the lowest point of the canal in the seated position, the subsequent recline may not generate sufficient endolymph flow to displace the particles and trigger nystagmus, which could account for some of our LDT-negative cases. Therefore, while our study confirms the clinical utility of LDT in real-world practice, the underlying mechanism for its variable elicitation rate requires further investigation. Future prospective studies should focus on the initial position of otoconia using more refined positional assessments.

A key finding of this study is that among canalithiasis patients, the fLDT-positive group required significantly fewer diagnostic steps than the fLDT-negative group. Given that the SRT often provokes intense vertigo and nausea, limiting patient tolerance for repeated maneuvers, and that multiple rolls are frequently needed to determine lateralization, a positive LDT can markedly simplify the diagnostic process, reduce patient discomfort, and allow earlier initiation of repositioning therapy. Therefore, we contend that a positive LDN offers considerable practical benefit in streamlining the management of canalithiasis HC-BPPV. This advantage was not observed in the cupulolithiasis subgroup, which may be attributed to the smaller sample size (*n* = 47) —a matter warranting further investigation in larger cohorts.

Although the LDT does not yield a 100% positivity rate, its relatively lower elicitation rate, particularly in canalithiasis, may be explained by several mechanisms: when otoconia are already near the lowest point of the canal in the supine position, recline may not generate sufficient endolymph flow; additionally, otoconia may be more dispersed, producing an inadequate stimulus. To explore factors influencing LDT results, we compared nystagmus characteristics between fLDT-positive and fLDT-negative groups. In canalithiasis patients, the fLDT-positive group exhibited greater intensity (*p* = 0.003) and shorter latency (*p* = 0.003) of the first leftward-roll nystagmus. This finding has important clinical implications: First, the initial roll captures the most authentic pathological state. At this moment, the endolymphatic system is in a resting, undisturbed state, and the induced nystagmus most truly reflects the natural position of the otoconia and the sensitivity of the semicircular canal. Subsequent rolls may show attenuated responses due to adaptation or micro-displacement of the otoconia. The greater intensity and shorter latency observed in the fLDT-positive group suggest that when the LDT does elicit a response, it identifies a distinct subgroup of canalithiasis patients with more “irritable” characteristics—otoconia that are likely more aggregated or positioned closer to the cupula, generating stronger endolymph flow upon positional change; Second, the characteristics of the initial roll may aid in the early differentiation of BPPV subtypes. This phenomenon of enhanced intensity during the first leftward roll was observed only in patients with canalolithiasis, whereas in patients with cupulolithiasis, the fLDT-positive group only showed shorter latency without differences in nystagmus intensity. This discrepancy suggests that significantly enhanced intensity during the initial roll is more indicative of canalolithiasis, while isolated latency shortening may point toward cupulolithiasis, offering a new perspective for early subtype differentiation. Thus, fLDT positivity may serve as a marker for a more active or mechanically responsive form of BPPV, providing insights into the heterogeneous nature of its pathophysiology. In cupulolithiasis patients, only shorter latency reached statistical significance (*p* = 0.031). Furthermore, multivariate logistic regression identified older age (≥60 years) as an independent predictor of a positive fLDT in the canalithiasis subgroup, suggesting that age-related inner ear changes—such as increased otoconial degeneration or reduced endolymph viscosity—may influence semicircular canal sensitivity to positional testing. This finding highlights the multifactorial nature of LDT response, where both mechanical factors (otoconia characteristics) and patient factors (age) contribute to test positivity.

Currently, there is limited research on the mechanisms underlying the variable positivity rate of LDT. Our findings indicate that age may be an independent factor affecting LDN elicitation in canalithiasis BPPV. However, the specific mechanisms by which age influences test positivity remain unclear and warrant further investigation. Separately from the age-related findings, we also observed that LDT-positive patients exhibited shorter nystagmus latency and greater nystagmus intensity. We speculate that the hair cells in these patients may exist in a more “vulnerable and hypersensitive” state, with a lower threshold and more rapid, intense response to gravitational stimuli, manifesting as shortened latency and enhanced intensity. Further studies are needed to validate this hypothesis and elucidate the underlying mechanisms.

## 5 Limitations

First, this was a single-center retrospective analysis. Second, while nystagmus characteristics were collected, comprehensive historical clinical data were limited, and systematic follow-up beyond the 30-min post-treatment assessment was not performed. Third, the sample size for the cupulolithiasis HC-BPPV subgroup was relatively small, and we did not further subclassify the exact location of otoconia within the horizontal canal, including the utricular side where atypical nystagmus may occur and potentially lead to diagnostic errors. Future studies should employ a prospective design, comprehensively collect historical and clinical data, include systematic follow-up (e.g., at 24 h, 1 week, and 1 month), aim for larger sample sizes, and incorporate more detailed subclassification of otoconia location.

## 6 Conclusion

In summary, the present study confirms that the LDT demonstrates an overall positivity rate of 60.3%, with the fLDT yielding a rate of 44.5%, results that align with the majority of existing literature. Importantly, LDT exhibits a high lateralization accuracy of 96.7%, supporting its reliability as a diagnostic tool for HC-BPPV. Given its favorable patient tolerance and procedural simplicity, we recommend incorporating LDT as a routine adjunct to the SRT in clinical practice. Furthermore, our findings identify advanced age (≥60 years) as a potential factor associated with a higher likelihood of eliciting LDN in patients with canalithiasis HC-BPPV. Nystagmus intensity and latency emerged as key parameters that may help distinguish LDT-positive from LDT-negative responses. These insights not only enhance our understanding of the test’s variability but also offer valuable directions for future research into the pathophysiological mechanisms underlying LDN in HC-BPPV.

## Data Availability

The raw data supporting the conclusions of this article will be made available by the authors, without undue reservation.
